# Possible Prognostic Potential of RANKL and OPG in Metastatic Breast Cancer Egyptian Females

**DOI:** 10.31557/APJCP.2020.21.2.355

**Published:** 2020

**Authors:** Olfat Gamil Shaker, Eman Maher Elbaz

**Affiliations:** 1 *Department of Medical Biochemistry and Molecular Biology, Faculty of Medicine, *; 2 *Department of Biochemistry, Faculty of Pharmacy, Cairo University, Cairo, Egypt. *

**Keywords:** Breast cancer, RANKL, OPG, YKL-40

## Abstract

**Objectives:**

Searching for sensitive, minimally invasive biomarkers that represent tumor-associated changes in the peripheral blood might enable the early diagnosis of breast cancer (BC) and monitoring of tumor progression.

**Methods:**

Herein, we investigated the association of some circulating biomarkers with the risk of metastasis. In the current study, 115 BC patients which were subdivided into two groups: nonmetastatic breast cancer patients (NMBC) (n=83) and metastatic breast cancer patients (MBC) (n=32), and 79 apparently healthy controls were recruited. Serum protein levels of lysosomal protein transmembrane 4 beta (LAPTM4B), receptor activator of nuclear factor-kappa b (NF-Kb) ligand (RANKL), osteoprotegerin (OPG), vitamin D (VIT D), chitinase-3-like protein 1 (also known as YKL-40), and sirtuin 1 (SIRT1) were assessed in blood samples using ELISA technique.

**Results:**

The results showed that RANKL and OPG had the highest diagnostic potential for MBC detection, with area under the curve values of 0.97 and 0.94, respectively. Moreover, logistic regression analysis showed that RANKL had the highest differentiation power in the discrimination of MBC from NMBC.

**Conclusion:**

The study highlighted that measuring RANKL and OPG may be helpful in the early detection of metastasis in Egyptian patients with BC.

## Introduction

Breast cancer (BC) is the leading cause of mortality among women worldwide (Jemal and Jemal, 2010). In Egypt, BC is the most common malignancy in women accounting for 32 % of female cancer (Ibrahim et al., 2014). Moreover, there are approximately 23081 new BC cases among women in 2018, according to GLOBOCAN estimates of incidence (Bray et al., 2018). The etiology likely encompasses both hereditary and environmental risk factors (Mavaddat et al., 2010). BC begins as a confined disease but can migrate to the lymph nodes and metastasize to the lung, liver, brain, and bone (Weigelt et al., 2005). Therefore, effective biomarkers that can predict the prognosis of BC are urgently needed.

Any single marker has very limited clinical application for the detection of early-stage disease. However, the efficient detection of multiple biomarkers together would be more effective for determining the prognosis of patients with metastatic BC than using a single marker. Lysosomal protein transmembrane 4 beta (LAPTM4B), receptor activator of nuclear factor-kappa b (NF-Kb) ligand (RANKL), osteoprotegerin (OPG), vitamin D (VIT D), chitinase-3-like protein 1 (also known as YKL-40), and sirtuin 1 (SIRT1), have been reported to be upregulated in many human malignancies, including BC (Johansen et al. 2009; Weichhaus et al. 2015; Zhang et al. 2015).

LAPTM4B was first recognized as hepatocellular carcinoma (HCC)-associated gene (Zhang et al., 2001). Many studies have reported that both the gene and its protein product are upregulated in many human cancers (Liu et al., 2009), such as ovarian cancer (Yang et al., 2008) and extrahepatic cholangiocarcinoma (Zhou et al., 2008).

The receptor activator of nuclear factor NF-κB ligand (RANKL) has been linked with the increase in osteoclast-induced bone resorption, while osteoprotegerin (OPG) halts RANKL and hinders bone resorption. The balance of OPG/RANKL interactions is an essential regulator of osteoclastogenesis and bone turnover. In many cancer types, e.g., multiple myeloma, disruption of the RANKL/OPG ratio leads to RANKL triggering increased osteoclastogenesis (Sfiridaki et al., 2011). Remarkably, both osteoclastogenesis proteins have been reported to be increased in several types of cancers, including BC and multiple myeloma, showing their crucial role in tumor growth and dissemination (Sfiridaki et al., 2011; Infante et al., 2019). It has been reported that both RANKL and OPG involved in BC growth and progression (Rachner et al., 2019). 

YKL-40 is a member of the mammalian chitinase-like proteins (Johansen et al., 2009). It is a prominent protein in human synoviocytes, chondrocytes, and several cancer cell lines (Volck et al., 2001; Junker et al., 2005). Increased serum levels of YKL-40 have been reported in inflammation as well as in cellular development, differentiation, and angiogenesis (Recklies et al., 2002; Rathcke et al., 2009).

In addition to the role vitamin D plays in calcium homeostasis, it performs many immunogenic and antiproliferative activities in the body (Khan et al., 2011) by binding to vitamin D receptor (VDR) , which is found in various tissues and cells of the body, such as breast cells that have VDRs in their nucleus (Lowe et al., 2005; Giovannucci, 2005). It has been reported that low serum levels of vitamin D are common in diagnosis and linked to poor prognosis of BC (Goodwin et al., 2009).

SIRT1 is 1 of 7 members of the sirtuin family of nicotinamide adenine dinucleotide (NAD+)-dependent class III histone deacetylases (Chen et al., 2012). SIRT1 is a multifunctional protein that plays a crucial role in multiple pathways. SIRT1 has been reported to be upregulated in many human malignancies, including BC (Sung et al., 2010). In contrast, Wang et al., (2008) found that SIRT1 expression was decreased in many other types of cancers, such as glioblastoma, bladder carcinoma, prostate carcinoma, and ovarian cancers, compared to matching normal tissues.

This study aimed to investigate the potential association between serum levels of LAPTM4B, RANKL, OPG, YKL-40, VIT D, and SIRT1 and the risk of MBC among Egyptian women. We also examined their association with clinicopathological parameters and their potential as non-invasive prognostic biomarkers of BC.

## Materials and Methods

*Subjects and methods*

*Patients*

The current case study included 115 Egyptian female BC patients based on taking a full history and performing a clinical check up. Mammography and surgical biopsies were also carried out for additional clinical confirmation. The BC group was further divided into nonmetastatic breast cancer (NMBC) and metastatic breast cancer (MBC) groups. All patients were recruited from the Surgery Department, Faculty of Medicine, Cairo University. Shortly, the inclusion criteria encompassed the following: adult women, age range 20–70 years, and no previous treatment with chemo- and/or radiotherapy.

Apparently healthy women age matched to the patients’ population and with no family history of BC were recruited in the study as a control group.

The subjects were divided into the following four groups: Group I: (n=79) healthy women as a control group; Group II: (n=83) patients with NMBC; and Group III: (n=32) patients with MBC. The cancer cases were classified according to the tumor, node, metastasis (TNM) grading system: stage II included 19 cases; stage III included 64 cases; and stage IV included 32 cases. The clinicopathological data of all participants were supplied from their medical reports. All patients and controls gave their informed written consent in accordance with the ethical guidelines of the Helsinki Declaration (2000). This clinical study was approved by the ethics committee of the Faculty of Pharmacy, Cairo University (approval number: BC 2117).

*Sample collections and laboratory assays*

Peripheral blood samples from all study participants were withdrawn by trained technicians and collected in dry sterile tubes. The blood was left to clot and then centrifuged at 1,400 X g for 5 minutes in order to separate the serum, which was stored immediately at -20ºC till analysis. 

The serum was used for the determination of the following biomarkers; LAPTM4B, RANKL, OPG, YKL-40, VIT D, SIRT1 using Human specific enzyme-linked immunosorbent assay (ELISA) kits supplied from MyBioSource Inc.(San Diego, CA, USA) with the catalog numbers (Cat.#): MBS9332479, MBS268235, MBS267842, MBS177225, MBS267183, and MBS2503120, respectively. All measurements were done according to the manufacturer’s instructions. The measurements were performed at 450 nm.

*Statistical analysis*

Values were expressed as median (25^th^ – 75^th^ percentile) or as the number (percentage) when applicable. Qualitative data were compared using the chi-squared test. A Comparison between more than two groups was performed using Kruskal–Wallis ANOVA followed by Dunn’s multiple comparison test for quantitative data. Correlations were assessed by Spearman correlation analysis. The diagnostic performance of biomarkers was evaluated by receiver operating characteristic (ROC) analysis. The area under the curve (AUC), sensitivity, and specificity were also determined. Logistic regression analysis was also performed to predict BC and MBC. For all experiments, P < 0.05 was considered statistically significant. The statistical analysis was performed using GraphPad Prism 5 software.

## Results

*Characteristics of the study groups*

Demographic and clinicopathological characteristics of all the study groups are shown in Table 1. NMBC and MBC groups differed markedly in age (y), pathological status, family history, tumor size, and TNM stages (p< 0.0001). Moreover, both groups have differed significantly in tumor type (p= 0.009), as well as ER/PR hormonal status (p= 0.047).

*Serum biomarkers concentrations*

Concentrations of serum biomarkers are represented as the median (25 % -75 % percentile) in all the study groups (Table 2). LAPTM4B, RANKL OPG, YKL-40, and SIRT1 in serum showed a significant change in both cancer groups compared with control. While, VIT D serum concentration differed significantly in the MBC group only as compared with the control group. Intriguingly, the serum concentrations of RANKL and OPG in the two cancer groups exhibited a significant alteration in the MBC group compared with the NMBC group. On the other hand, serum LAPTM4B, YKL-40, and SIRT1 did not show marked change, indicating that they could not be used as markers to predict the MBC.

*Correlation between the studied biomarkers and clinical data*

Regarding the NMBC group, Table 3 revealed that VIT D serum concentration was associated with hypertension (P = 0.04). Moreover, there was a significant correlation between LAPTM4B and tumor size (P = 0.001) as well as between YKL-40 and TNM staging (P= 0.008) in the same group. Another part of our results, shown in Table 4, revealed that both RANKL and OPG were associated with tumor size (P = 0.03 in both) in the MBC group. LAPTM4B was also correlated with age (P = 0.02) in the same group.

*Correlation between the studied biomarkers*

Table 5 revealed that OPG showed (r=0.62 and 0.26, respectively) significant positive correlation with RANKL and YKL-40 (r=0.62 and 0.26, respectively). YKL-40 correlated positively with RANKL and SIRT1 (r=0.26 and 0.24, respectively). However, we observed negative significant correlation between OPG with LAPTM4B and VIT D (r= - 0.2 and - 0.21, respectively).

*Diagnostic interpretation of all studied biomarkers*

ROC analysis was performed to assess the diagnostic potential of all studied biomarkers to discriminate between BC patients with and without metastasis. We observed that RANKL showed the largest AUC of 0.97 (95% CI = 0.948 – 0.999, P < 0.0001, sensitivity = 96.4%, specificity = 84.4%, a cut off value < 180), followed by OPG, whose AUC was 0.94 (95% CI = 0.90 – 0.99, P < 0.0001, sensitivity = 90%, specificity = 93%, a cut off value < 123). Additionally, the results showed that YKL-40, VIT D, and SIRT1 may be useful to a lesser extent in this aspect (AUC = 0.68, 0.64, and 0.65, respectively). However, LAPTM4B was not effective alone as a potential diagnostic biomarker to differentiate between MBC and NMBC (AUC= 0.59). RANKL and OPG showed the highest differentiation potential. Using logistic regression analysis, we found that LAPTM4B and YKL-40 could be used together for the early detection of patients with BC, with R^2^=0.971 and percentage correction = 98%. However, only RANKL could be used to discriminate MBC, with R2= 0.802 and percentage correction = 92%.

**Table 1:** Clinical Characteristics of the Study Groups

	Control (n=79)	Non-metastatic breast cancer (NMBC) (n=83)	Metastatic breast cancer (MBC) (n=32)	*P*
Age, (y)				
≤ 40	78.50%	12%	20%	P< 0.0001
> 40	21.50%	88%	80%	
Pathological status				
I-Diabetes				P< 0.0001
No	100%	81%	77.50%	
Yes	0	19%	22.50%	
II-Hypertension				P< 0.0001
No	100	76%	77.50%	
Yes	0	24%	22.50%	
Family history				P< 0.0001
No	100	49.20%	28.10%	
Yes	0	50.80%	71.90%	
Tumor type				P= 0.009
Invasive ductal		91.60%	78.10%	
Invasive lobular		7.40%	21.90%	
Tumor size				P< 0.0001
< 5 cm		66%	93.80%	
> 5 cm		34%	6.20%	
TNM stages				P< 0.0001
II		23%	0	
III		77%	0	
IV		0	100%	
Hormonal status				P= 0.047
- ve ER/PR		41%	55%	
+ ve ER/PR		59%	45%	

**Table 2:** Serum Concentrations of Clinical Biomarkers in the Study Groups

Parameters Median (25^th^ – 75^th^ percentile)	Groups		
	Control	Non-metastatic breast cancer (NMBC)	Metastatic breast cancer (MBC)
LAPTM-4B (pg/ml)	392.5 (257-507.1)	1234 (873.5-2154) ^a^	1038 (794.8-1391) ^a^
RANKL (pg/ml)	44.45 (37.8-50.1)	47.18 (42.77-73.42) ^a^	270.8 (211.8-389.9)^ ab^
OPG (pg/ml)	15.6 (21.5-21.6)	53.2 (34.1-83.5) ^a^	174.4 (159.8-196.4) ^ab^
YKL-40 (ng/ml)	20.4 (18.8-28.3)	101.4 (49.4-186.9) ^a^	158.8 (143.3-186.9) ^a^
vit.D (nmol/l)	61.6 (51.5-79.6)	60.5 (38.6-75.3)	41.4 (40.7-85.9) ^a^
SIRT1(ng/ml)	4.2 (3.8-4.9)	8.1 (7.4-8.6) ^a^	8.6 (7.8-9.2) ^a^

Data represent as median (25th -75th percentile) a: Significantly different from the control group at P <0.05; b: significantly different from the the NMBC group at P <0.05. Abbreviations: LAPTM4B, Lysosomal protein transmembrane 4 beta; RANKL, receptor activator of nuclear factor NF-κB ligand; OPG, osteoprotegerin; YKL-40, human cartilage-glycoprotein; VIT D, vitamin D; SIRT1, sirtuin 1.

**Table 3:** Correlation between Serum Levels of LAPTM4B, RANKL, OPG, YKL-40, VIT D, and SIRT1 and the Clinicopathological Features of NMBC Patients

	LAPTM4B		RANKL		OPG		YKL-40		VIT D		SIRT1	
	r	*P*	r	*P*	r	*P*	r	*P*	r	*P*	r	*P*
Age	0.2	0.09	-0.14	0.2	0.07	0.5	-0.06	0.6	-0.11	0.4	0.07	0.5
Diabetes	-0.17	0.2	0.11	0.4	0.1	0.4	0.07	0.6	-0.15	0.2	0.1	0.4
Hypertension	-0.01	0.9	-0.11	0.3	-0.03	0.8	0.01	0.9	-0.23	***0.04***	-0.01	0.9
Family history	0.03	0.8	0.07	0.5	-0.13	0.3	-0.15	0.2	-0.06	0.6	-0.02	0.9
Tumor type	-0.2	0.08	-0.01	0.9	0.13	0.24	0.14	0.2	0.19	0.09	-0.15	0.17
Tumor size	0.4	***0.001***	-0.05	0.7	0.16	0.2	0.06	0.6	0.06	0.6	-0.15	0.2
TNM staging	0.17	0.1	0.12	0.3	0.12	0.3	-0.3	***0.008***	-0.01	0.9	0.004	0.9
ER/PR	0.09	0.5	-0.07	0.6	-0.15	0.2	0.16	0.1	0.01	0.9	0.014	0.9

Correlation between serum marker levels and clinicopathological features was evaluated by Spearman test at P < 0.05. P values in bold and italics are considered significant. Abbreviations: r, correlation coefficient; LAPTM4B, Lysosomal protein transmembrane 4 beta; RANKL, receptor activator of nuclear factor NF-κB ligand; OPG, osteoprotegerin; YKL-40, human cartilage-glycoprotein; VIT D, vitamin D; SIRT1, sirtuin 1.

**Figure 1:**
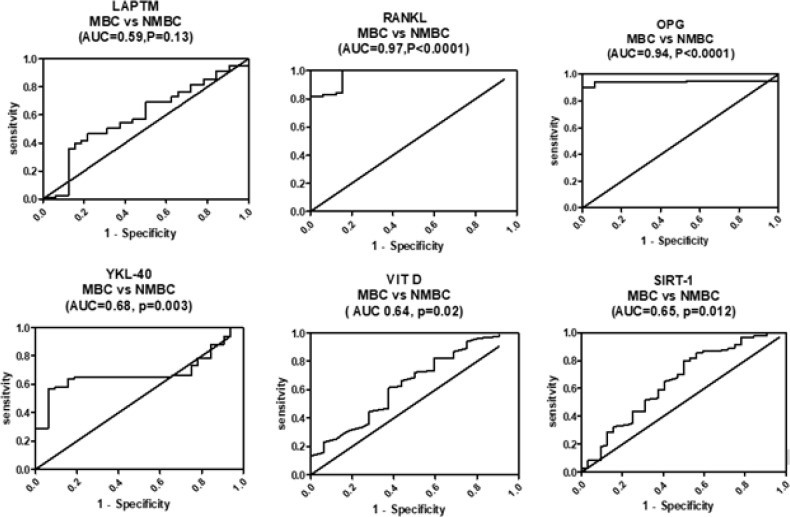
ROC Curve Analysis of Serum LAPTM4B, RANKL, OPG, YKL-40, VIT D, and SIRT1 Levels as Diagnostic Biomarkers that Distinguish between the MBC and the NMBC Patients

**Table 4:** Correlation between Serum Levels of LAPTM4B, RANKL, OPG, YKL-40, VIT D, and SIRT1 and the Clinicopathological Features of MBC Patients

Parameters	LAPTM4B		RANKL		OPG		YKL-40		VIT D		SIRT1	
	r	*P*	r	*P*	r	*P*	r	*P*	r	*P*	r	*P*
Age	0.42	***0.02***	0.03	0.9	0.16	0.4	0.07	0.7	-0.13	0.5	-0.3	0.1
Diabetes	-0.24	0.17	-0.24	0.2	-0.23	0.2	-0.13	0.5	-0.02	0.9	0	1
Hypertension	0.03	0.9	-0.15	0.4	-0.15	0.4	-0.03	0.9	0.14	0.4	-0.27	0.1
Tumor type	-0.22	0.23	-0.004	0.9	-0.09	0.6	0.08	0.7	-0.15	0.4	0.1	0.6
Tumor size	-0.11	0.54	-0.392	***0.03***	-0.38	***0.03***	0	1	-0.05	0.8	-0.2	0.2
TNM staging	-0.3	0.15	-0.2	0.4	-0.2	0.2	-0.06	0.8	0.2	0.2	-0.09	0.6
ER/PR	-0.004	0.9	0.16	0.4	-0.2	0.3	0.02	0.9	0.23	0.1	-0.01	0.6

Correlation between serum marker levels and clinicopathological features was evaluated by Spearman test at P < 0.05. P values in bold and italics are considered significant. Abbreviations: r, correlation coefficient; LAPTM4B, Lysosomal protein transmembrane 4 beta; RANKL, receptor activator of nuclear factor NF-κB ligand; OPG, osteoprotegerin; YKL-40, human cartilage-glycoprotein; VIT D, vitamin D; SIRT1, sirtuin 1.

**Table 5:** Correlation between Serum Biomarker Levels in All Study Groups

Parameters	LAPTM4B		RANKL		OPG		YKL-40		VIT D		SIRT1	
	r	*P*	r	*P*	r	*P*	r	*P*	r	*P*	r	*P*
LAPTM-4B			-0.13	0.18	-0.2	***0.03***	-0.06	0.6	0.01	0.9	-0.09	0.3
RANKL	-0.13	0.18			0.62	***0.0001***	0.26	***0.005***	-0.11	0.25	0.17	0.07
OPG	-0.2	***0.03***	0.62	***0.0001***			0.26	***0.005***	-0.21	***0.02***	0.17	0.06
YKL-40	-0.06	0.6	0.26	***0.005***	0.26	***0.005***			0.14		0.24	***0.01***
VIT D	0.01	0.9	-0.11	0.25	-0.21	***0.02***	0.14	0.14			-0.16	0.09
SIRT1	-0.09	0.3	0.17	0.07	0.17	0.06	0.24	***0.01***	0.16	0.09		

Correlations between parameters were determined by Spearman correlation analysis at p < 0.05. P values in bold and italics are considered significant. Abbreviations: r, correlation coefficient; LAPTM4B, lysosomal protein transmembrane 4 beta; RANKL, receptor activator of nuclear factor NF-κB ligand; OPG, osteoprotegerin; YKL-40, human cartilage-glycoprotein; VIT D, vitamin D; SIRT1, sirtuin 1.

## Discussion

BC is the most common malignant neoplasm among women worldwide (Patsialou et al., 2012). In Egypt, it is the most common malignancy among females (Ibrahim et al., 2014). However, metastasis is the main cause of BC mortality (Tarin, 2008). Although progress has been made in cancer treatment and the methods used to detect its progression, BC continues to be a major life-threatening disease among women (Youlden et al., 2014).

Currently, it is not recommended to use a single screening test for cancer. Therefore, many recent guidelines encourage the use of multiple screening tests to anticipate the disease and improve the clinical outcome. In our study, we assessed 6 biomarkers (LAPTM4B, RANKL, OPG, YKL-40, VIT D, and SIRT1), LAPTM4B was first identified as a hepatocellular carcinoma-related gene in mammals (Liu et al., 2003). In the present study, we demonstrated a significant elevation in serum LAPTM4B levels in both BC groups compared with the control. However, the difference in levels between the two cancer groups was not significant. A similar result was detected by Xiao et al., (2013), who reported that LAPTM4B was activated and overexpressed in serum of BC patients. Moreover, other studies have demonstrated that LAPTM4B has been upregulated in many human cancers, such as lung cancer (Tang et al., 2014), gastric cancer (Zhang et al. 2014), and prostate cancer (Zhang et al., 2015), and is known to have a role as a dynamic cancer-associated biomarker that is involved in several biological processes (Kasper et al., 2005).

We observed significant increases in both RANKL and OPG serum levels in the MBC group compared with the control and NMBC groups. Both RANKL and OPG showed the highest discriminatory potential to distinguish between BC patients with and without metastasis. This result is in accordance with the previous report of elevated serum levels of both biomarkers in patients with breast or lung cancer metastasis (Mountzios et al., 2007). Both RANKL and OPG have been shown to have a direct oncogenic role, as RANKL has a positive influence on the multiplication and endurance of mammary cells. It also promotes the mitogenic action of progesterone in the breast epithelium (Tanos et al., 2013). It has been reported that inhibition of RANKL suppressed tumor growth and proliferation (de Groot et al. 2018). In addition, OPG was reported to be overexpressed in human BC cell lines and tumor samples, showing its potential to promote breast tumorigenesis and tumour spreading (Weichhaus et al., 2015; Infante et al., 2019).

The present investigation also showed a significant elevation in serum YKL-40 levels in cancer groups compared with the control group. These data are in line with those reported by a previous study (Yamac et al., 2008). Although the biological role of YKL-40 remains obscure, Johansen et al., (2009) reported that serum YKL-40 is an important marker that is associated with poorer prognosis and shorter disease-free survival in several types of solid tumor, including breast, ovarian, uterine, colon, lung, and kidney ttumor. YKL-40 also plays a crucial role in the growth, differentiation, and angiogenesis of malignant cells (Hamilton et al. 2015).

In addition, the current study demonstrated that there was a significant decrease in serum 25-hydroxyvitamin D levels in the MBC group compared with healthy controls. Nonetheless, our results revealed that the lowest level of VIT D was observed in MBC. This finding is in agreement with many studies that suggested a defensive effect of VIT D against BC growth, invasion, and mortality (Bertone-Johnson et al., 2005; Imtiaz et al., 2012). 

25-Hydroxyvitamin D is the principal form of VIT D in the circulation and is the best indicator of the VIT D status of the body owing to its relatively long t1/2. Several explanations have been presented for the inhibitory effect of VIT D on BC cell growth. VIT D controls the phenotype of human BC cells, and the introduction of 25-dihydroxyvitamin D to cell culture results in an improvement in features accompanied by poor prognosis (Pendás-Franco et al., 2007). Moreover, VIT D inhibits breast tumor invasion and proliferation (Shao et al., 2012).

Notably, in this study, the serum concentration of SIRT1 was significantly higher in the BC groups, either NMBC or MBC, compared to the control group. Remarkably, MBC showed the highest level of serum SIRT1. Similar observations were made by Chen et al., (2015), who reported that SIRT1 overexpression can boost tumor progression and is associated with a poor prognosis in colorectal carcinoma patients. Nevertheless, SIRT1 is upregulated in a range of cancers, including lymphomas, leukemia, and soft tissue sarcomas, prostate cancer, and lung and colon carcinomas. Conversely, it has also been shown that SIRT1 can stimulate stress defense and DNA restoration mechanisms, enabling the protection of genomic integrity (Alcaín and Villalba, 2009).

Taken together, our data suggest that RANKL and OPG could be used as prognostic biomarkers for the detection of MBC in Egyptian women. Therefore, routine measurement of RANKL and OPG might be recommended to enable the early detection of MBC in BC patients.
